# High Fluorescence of Phytochromes Does Not Require Chromophore Protonation

**DOI:** 10.3390/molecules29204948

**Published:** 2024-10-19

**Authors:** Sagie Katz, Hoang Trong Phan, Fabian Rieder, Franziska Seifert, Markus Pietzsch, Jan Laufer, Franz-Josef Schmitt, Peter Hildebrandt

**Affiliations:** 1 Institute of Chemistry, Technical University Berlin, Sekr. PC14, Straße des 17. Juni 135, D-10623 Berlin, Germany; sagie.katz@tu-berlin.de; 2 Institute of Physics, Martin Luther University Halle-Wittenberg, von-Danckelmann-Platz 3, D-06120 Halle (Saale), Germany; hoang.phan@leibniz-inm.de (H.T.P.); fabian.rieder@student.uni-halle.de (F.R.); jan.laufer@physik.uni-halle.de (J.L.); 3 Leibniz Institute for New Materials, Campus D2 2, D-66123 Saarbrücken, Germany; 4 Institute of Pharmacy, Martin-Luther-University Halle-Wittenberg, Weinbergweg 22, D-06120 Halle (Saale), Germany; franziska.seifert@pharmazie.uni-halle.de (F.S.); markus.pietzsch@pharmazie.uni-halle.de (M.P.)

**Keywords:** phytochrome, fluorescence, Raman, tetrapyrrole, protonation

## Abstract

Fluorescing proteins emitting in the near-infrared region are of high importance in various fields of biomedicine and applied life sciences. Promising candidates are phytochromes that can be engineered to a small size and genetically attached to a target system for in vivo monitoring. Here, we have investigated two of these minimal single-domain phytochromes, miRFP670nano3 and miRFP718nano, aiming at a better understanding of the structural parameters that control the fluorescence properties of the covalently bound biliverdin (BV) chromophore. On the basis of resonance Raman and time-resolved fluorescence spectroscopy, it is shown that in both proteins, BV is deprotonated at one of the inner pyrrole rings (B or C). This protonation pattern, which is unusual for tetrapyrroles in proteins, implies an equilibrium between a B- and C-protonated tautomer. The dynamics of the equilibrium are slow compared to the fluorescence lifetime in miRFP670nano3 but much faster in miRFP718nano, both in the ground and excited states. The different rates of proton exchange are most likely due to the different structural dynamics of the more rigid and more flexible chromophore in miRFP670nano3 and miRFP718nano, respectively. We suggest that these structural properties account for the quite different fluorescent quantum yields of both proteins.

## 1. Introduction

**Figure 1:**
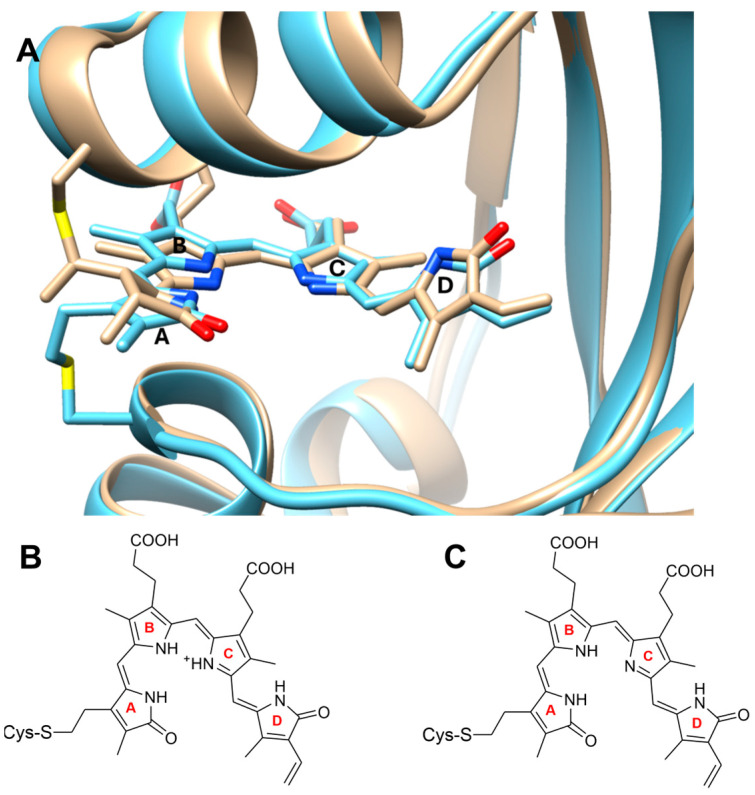
(**A**), Chromophore binding pockets of miRFP670nano3 (tan; PDB entry 6MGH) [13,28] and miRFP718nano (cyan, PDB entry 7LSD) [14,29]. Ring A is covalently attached to cysteine 57 and 86 in miRFP670nano3 and miRFP718nano, respectively. The structures of the biliverdin chromophores in the ZZZssa configuration are shown in the (**B**) protonated and (**C**) deprotonated form.

Concomitant to the progress in fluorescence microscopy and its applications in biology and medicine, increasing efforts have been made in the search for appropriate proteinic dyes that are also applicable for deep tissue investigations [1,2,3,4,5,6]. Important requirements are strong absorption bands in the red spectral region and high fluorescence quantum yields. In this respect, derivatives of the Green fluorescent protein (GFP) that contain a chromophore generated from amino acids are promising sensors as they can be (co)expressed in target cellular systems. However, GFP derivatives do not exceed 600 nm in absorption and 680 nm in emission, which is required for deep tissue imaging [7,8]. Therefore, proteins carrying open-chain tetrapyrroles are particularly interesting candidates since such chromophores exhibit more red-shifted absorption and fluorescence and are frequently produced by the target organisms themselves. Among these proteins, phytochromes have been widely explored in the past [4,6,9,10,11,12,13,14]. These photoreceptors have a strong electronic transition between 650 and 700 nm [15]. Natural representatives of this class of proteins exhibit a low fluorescence quantum yield of typically only 2% and undergo photoisomerization with an efficiency of ca. 15%. However, protein engineering *via* rational design and random mutagenesis succeeded in impairing photoconversion and increasing the fluorescence quantum yield up to nearly 20% [16]. For such infrared (IR) fluorescence-optimized bacterial phytochromes (iRFP), the applicability for deep tissue examinations has been documented [3,4,17]. These iRFPs consist of only two domains, the GAF (cGMP-specific phosphodiesterases, adenylyl cyclases, and FhlA) and PAS (Per-Arnt-Sim) domains, which are required to bind the biliverdin (BV) chromophore in a native-like manner. A further size reduction of the fluorescent protein was achieved upon focusing on a phytochrome subfamily, denoted as cyanobacteriochromes (CBCR). These CBCRs were discovered in cyanobacteria twenty years ago [18,19,20] and have been widely studied since then [21,22,23,24,25,26]. They typically include several GAF domains bind PCB, although recently, Fushimi et al. succeeded in engineering a CBCR (AnPixJg2) to accept BV as a chromophore [27], which—unlike PCB—is produced in mammalians. Based on this knowledge, Verkusha and coworkers generated a single GAF domain binds BV [13,14]. In those minimal iRFPs (miRFPnano), the considerable size reduction to ca. 17 kDa offers advantages for using the fluorescent probe as a tag to target proteins. For two miRFPnanos, miRFP670nano3 and miRFP718nano, the three-dimensional structures have been determined [13,14,28,29]. The structures of the chromophores, exhibiting a ZZZssa configuration, and the binding pockets are very similar (Figure 1A), and, as far as the crystal structure allows drawing a conclusion, are closely related to those of other fluorescent phytochromes, such as iRFP713. The sensors are bright, exhibiting very high fluorescence quantum yields of 5.6% (miRFP718nano) and 18.5% (miRFP670nano3) [28,29,30].

In this work, we have studied these two proteins by resonance Raman (RR) spectroscopy, complemented by time-resolved fluorescence (TRF) spectroscopy. The objective of the work was to elucidate the structural details of the chromophore (Figure 1B,C), which control nonradiative and radiative excited state decay channels. One advantage of RR spectroscopy roots in the sensitivity of vibrational spectroscopy towards protonation states and hydrogen bonding [31], which are not accessible from the X-ray structures with the present crystallographic resolution. Spectrally resolved TRF allows for the probing for conformations with different fluorescence yields. Thus, the combination of both techniques provides insight into the correlation between the protonation pattern and fluorescence quantum yield, as well as the proton dynamics of the fluorescent proteins.

## 2. Results

**Figure 2:**
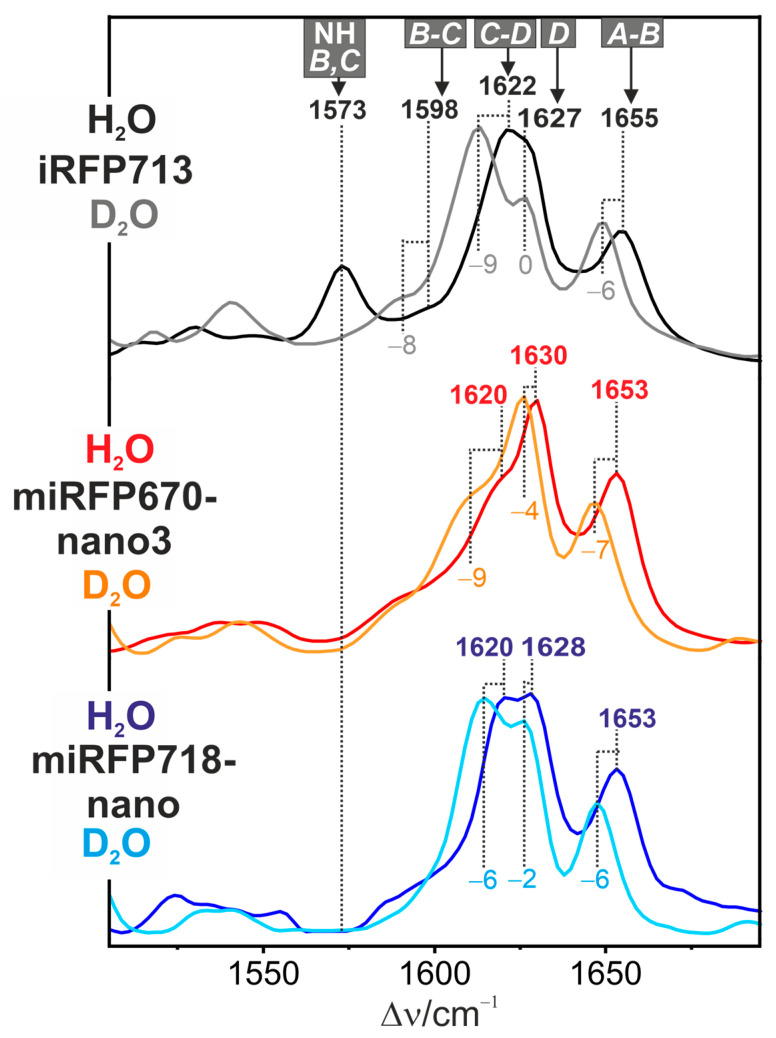
RR spectra of iRFP713 (black, top; taken from ref. [32]), miRFP670nano3 (red, middle), and miRFP718nano (blue, bottom) in H_2_O (pH 7.4). The corresponding spectra in D_2_O (pD 7.4) are displayed in grey, orange, and cyan, respectively. All spectra were measured at 90 K with 1064 nm excitation.

We have compared the RR spectra of miRFP670nano3 and miRFP718nano with those of the benchmark iRFP713 derived from the PAS-GAF domain of the bacteriophytochrome P2 from *Rhodopseudomonas palustris* (*Rp*BphP2), which has been extensively studied previously [30,32,33]. The most important information can be obtained from the spectral region between 1500 and 1700 cm^−1^, which is diagnostic of the tetrapyrrole configuration and conformation as well as the pyrrole nitrogen protonation state (Figure 2) [31].

**Figure A1:**
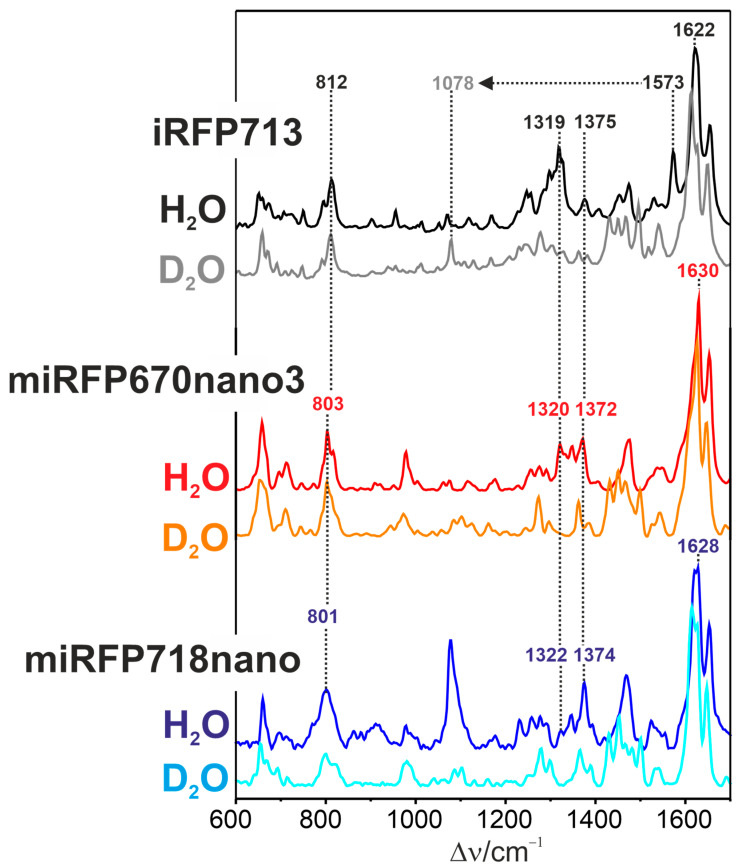
RR spectra of iRFP713 (black, top), miRFP670nano3 (red, middle) and miRFP718nano (blue, bottom) in H_2_O (pH 7.4). The corresponding spectra in D_2_O (pD 7.4) are displayed in grey, orange, and cyan, respectively. All spectra were measured at 90 K with 1064 nm excitation.

The strongest bands in this region originate from modes with large contributions from the stretching coordinates of the three methine bridges coupled to the N-H bending of the adjacent pyrrole groups. In iRFP713, these modes have been readily assigned to the A-B, C-D, and B-C stretchings at 1655, 1622, and 1598 cm^−1^, respectively. Their frequencies are characteristic of the ZZZssa configuration of the Pr state. Also, the frequency shifts of these modes upon H/D exchange are typical of the Pr chromophore in bacteriophytochromes (Figure 2 and Figure A1) [31]. In addition, these shifts reveal that the most prominent peak at 1622 cm^−1^ is composed of two closely spaced components; one of them is the H/D sensitive C-D stretching mode, whereas the second one at 1627 cm^−1^ remains unaffected upon H/D exchange and is attributed to a C=C stretching mode localized in ring D. The four stretching modes are accompanied by the spectral marker of the protonation state at 1573 cm^−1^ [31,32,34]. This mode is essentially a pure N-H in-plane bending (NH ip) of rings B and C, as reflected by the downshift to ca. 1078 cm^−1^ in D_2_O, and thus represents an unambiguous indicator of the protonated (cationic) form of tetrapyrroles (Figure 1B). Its frequency can vary by less than 10 cm^−1^ among the various Pr states of phytochromes, reflecting a moderate sensitivity to the hydrogen-bonding interactions.

In both miRFP670nano3 and miRFP718nano, the BV chromophore adopts the same ZZZssa configuration as in iRFP713, such that very similar RR spectra are expected. However, first, the NH ip mode is missing in both miRFP670nano3 and miRFP718nano (Figure 2), implying that the inner rings B and C carry only one proton (neutral form) (Figure 1C). Second, the C=C stretching region is dominated by two bands between 1600 and 1630 cm^−1^, which, unlike iRFP713, are both slightly H/D sensitive (Figure 2). This is not surprising since neutral and protonated tetrapyrroles are different molecules with distinct normal mode distributions and compositions. In miRFP670nano3 and miRFP718nano, the relative intensities of the bands between 1600 and 1640 cm^−1^ are different, which may be interpreted in terms of different localizations of the proton on either ring B or ring C. Following a previous study on the Meta-Rc intermediates of phytochromes carrying a deprotonated chromophore [35], the doublet reflects a tautomeric equilibrium with the high and low-frequency components originating from the tautomers with protonated B and C rings, respectively. Accordingly, the proton seems to reside mainly on ring B in miRFP670nano3 but in roughly equal proportions on rings C and B in miRFP718nano. The interpretation of different tautomeric equilibria is in line with the RR spectroscopic analysis of the Pg state of the CBCR RcaE, which carries a deprotonated PCB chromophore in the ZZZssa configuration [23]. Based on AnPixJg2 models, including a B- and C-protonated chromophore, the authors employed quantum mechanical/molecular mechanical methods to calculate the respective Raman spectra. Upon comparison with the experimental RR spectrum, it was concluded that ring B was deprotonated. When these calculated spectra are compared with the RR spectra of miRFP670nano3 and miRFP718nano, it is quite obvious that none of the two calculated spectra alone is similar to the experimental spectra. Instead, superpositions of the calculated spectra are required for a satisfactory description, reflecting the coexistence of both tautomers, albeit with different relative weights in miRFP670nano3 and miRFP718nano.

**Figure 3:**
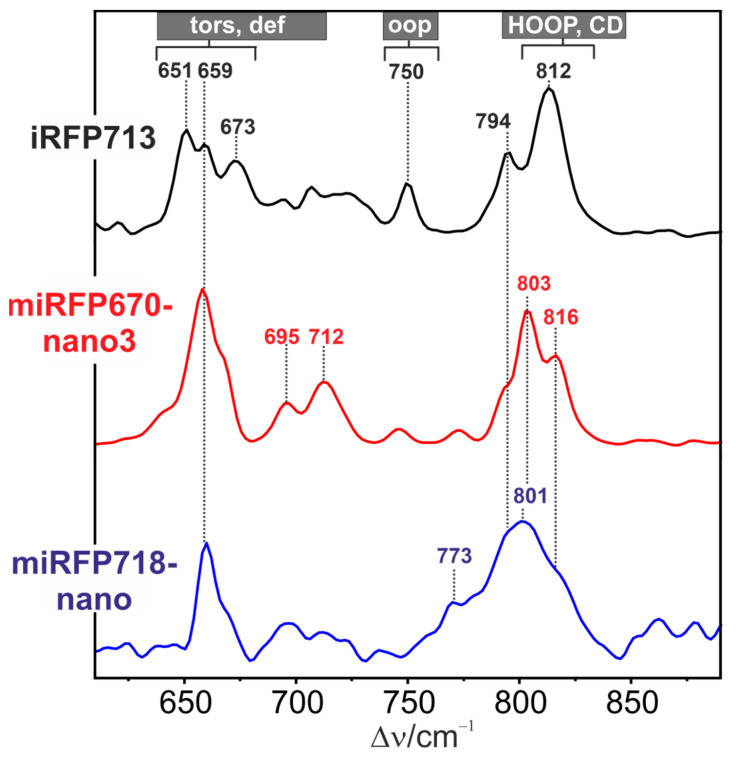
RR spectra of iRFP713 (black, top; taken from Ref. [32]), miRFP670nano3 (red, middle), and miRFP718nano (blue, bottom) in H_2_O (pH 7.4). All spectra were measured at 90 K with 1064 nm excitation.

The different distributions of the two tautomeric forms are also reflected by hydrogen-out-of-plane (HOOP) modes of the methine bridges, specifically of the C-D bridge (Figure 3). The position of this mode, which is typically observed between 800 and 820 cm^−1^, varies with the dihedral angles of the methine bridges [36]. In contrast to the single band at 812 cm^−1^ in iRFP713, we note two bands at 803 and 816 cm^−1^ in miRFP670nano3, probably reflecting the two tautomers since the protonation of ring B or ring C most likely affects the torsion of the C-D methine bridge. Accordingly, the situation is different in miRFP718nano, which displays a very broad signal covering the two C-D HOOP modes. This can be interpreted in terms of a fast proton transfer between the two sites, which may cause an exchange broadening of the two bands if the transfer occurs on the short ps time scale [37].

**Figure A2:**
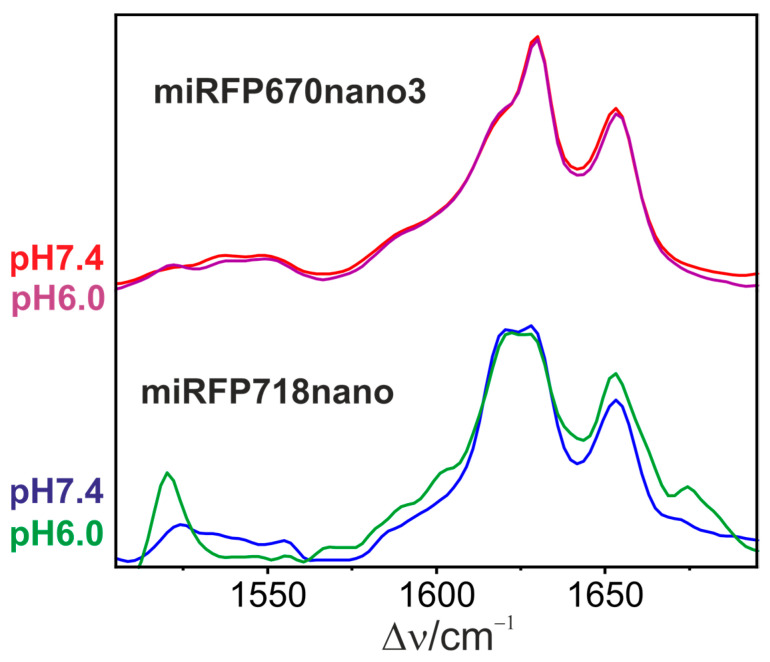
RR spectra of miRFP670nano3 (red) and miRFP718nano (blue) in H_2_O at pH 7.4 compared with the corresponding spectra at pH 6.0 displayed in magenta and green, respectively. All spectra were measured at 90 K with 1064 nm excitation.

**Figure A3:**
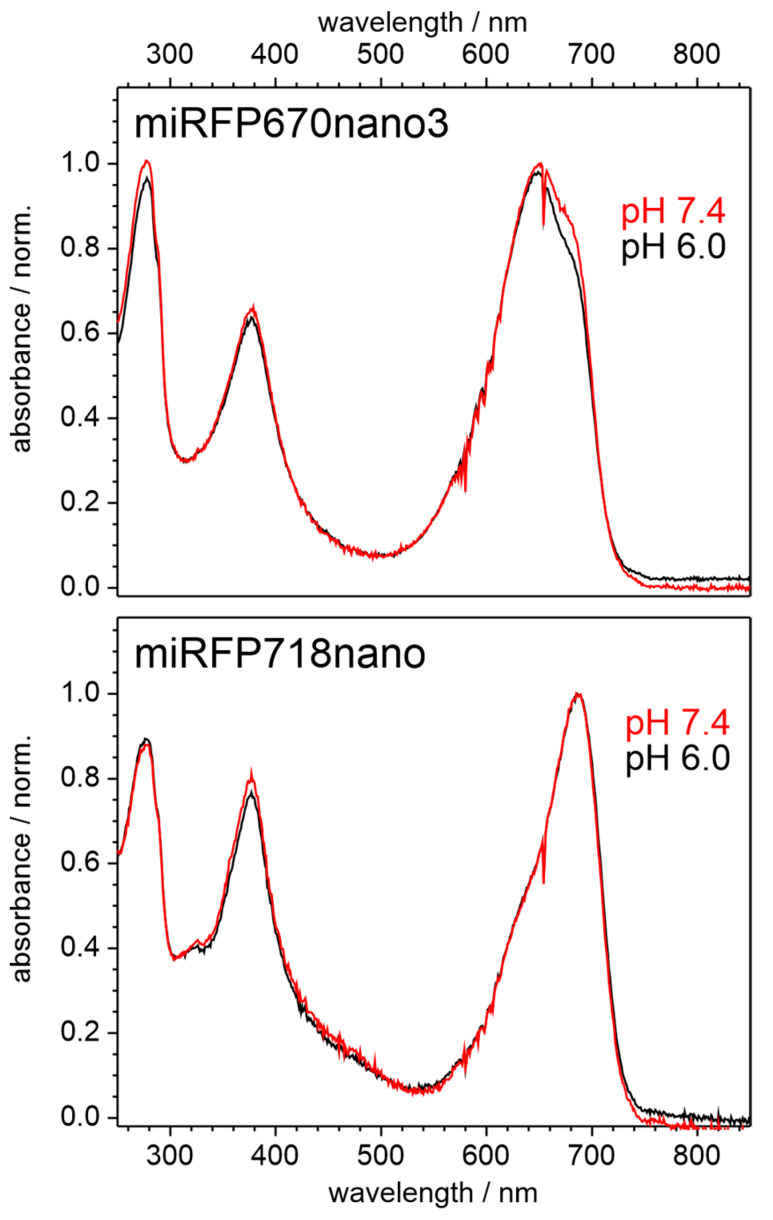
Absorption spectra of miRFP670nano3 (**top**) in H_2_O buffered with C3 buffer at pH 7.4 (red) and pH 6.0 (black). The (**bottom**) panel shows the absorption spectra of miRFP718nano at pH 7.4 (red) and pH 6.0 (black). The spectra are normalized to 1.

**Figure A4:**
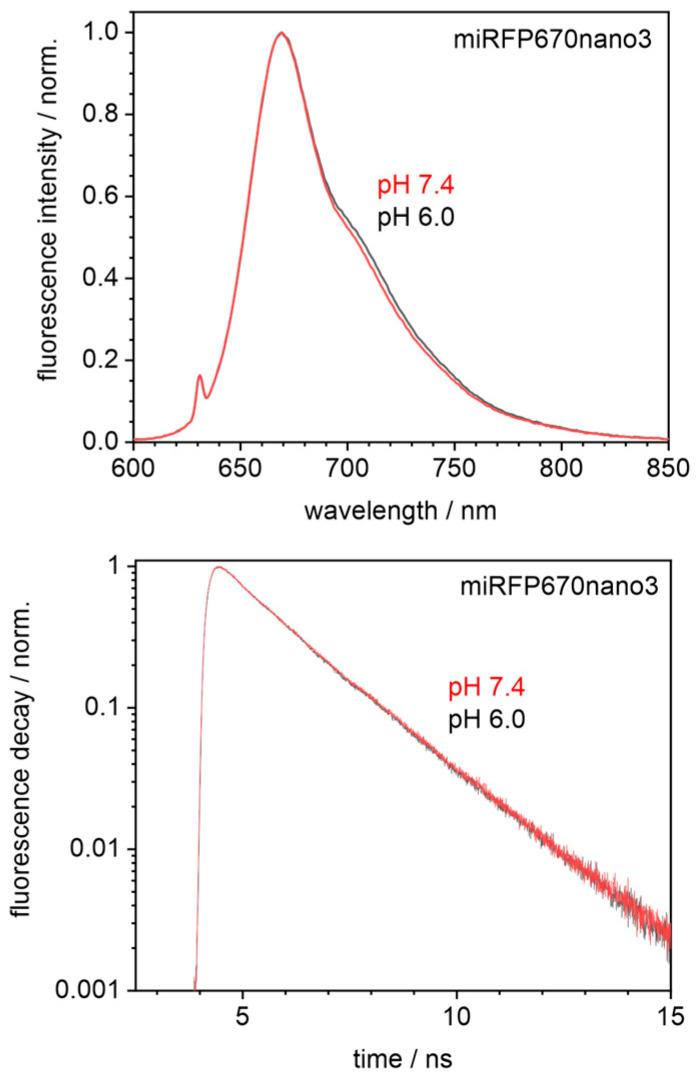
(**Top**): Fluorescence spectra of miRFP670nano3 at pH 7.4 (red) compared to pH 6.0 (black). The (**bottom**) panel shows the fluorescence decay of miRFP670nano3 in H_2_O buffered with C3 at pH 7.4 (red) compared with the corresponding spectra at pH 6.0 (black). The decay was registered in a 12.5 nm wide wavelength band centered at 669 nm after excitation with 632 nm laser light. The spectra and the fluorescence decay are normalized to 1.

**Figure A5:**
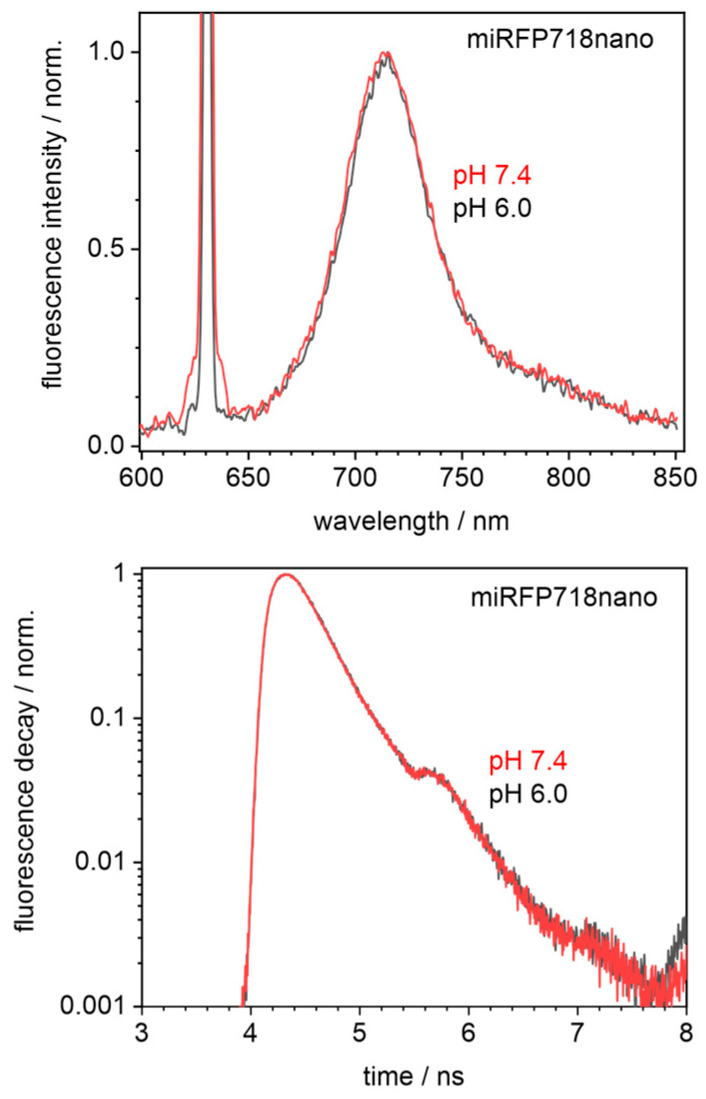
(**Top**): Fluorescence spectra of miRFP718nano at pH 7.4 (red) compared to pH 6.0 (black). The (**bottom**) panel shows the fluorescence decay of miRFP718nano in H_2_O buffered with C3 at pH 7.4 (red) compared with the corresponding spectra at pH 6.0 (black). The decay was registered in a 12.5 nm wide wavelength band centered at 669 nm after excitation with 632 nm laser light. The spectra and the fluorescence decay are normalized to 1.

The RR spectra remain unchanged upon acidification of the solution down to pH 6.0 (Figure A2). This is also true for the absorption and fluorescence spectra, including the fluorescence decay, which are essentially the same at pH 7.4 and 6.0 (Figure A3, Figure A4 and Figure A5). These findings show that in this pH range, there are no protonation reactions at the chromophore or in its immediate vicinity.

**Figure 4:**
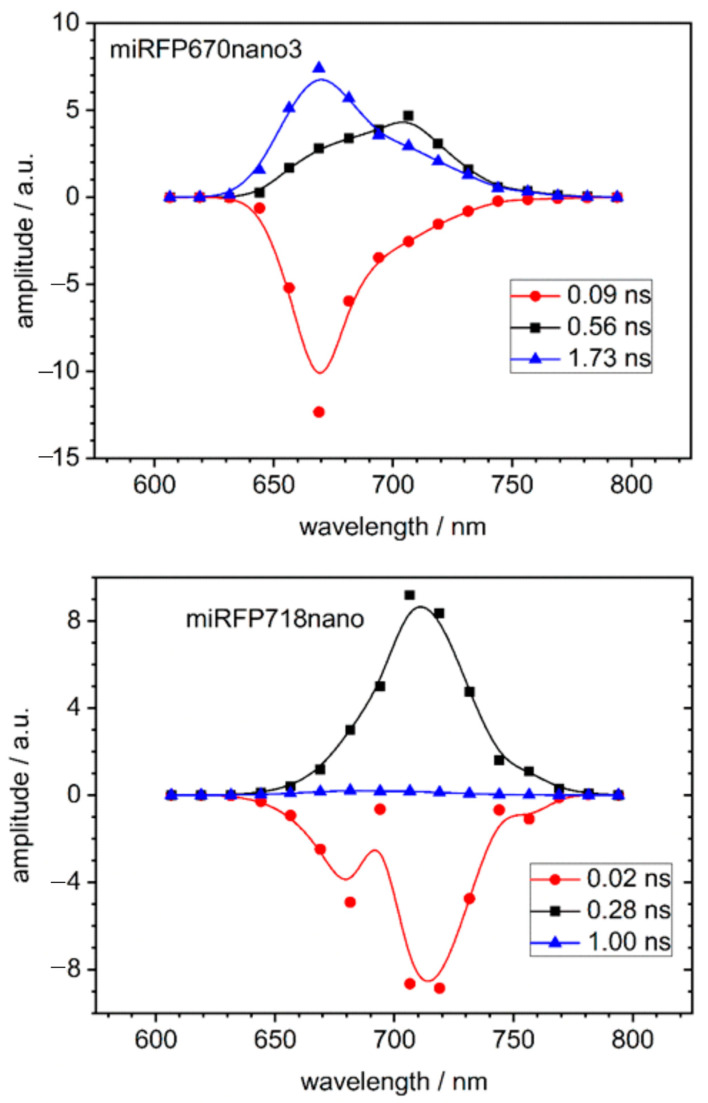
(**Top**): Decay associated spectra of miRFP670nano in H_2_O buffered with C3 at pH 7.4 compared with the corresponding spectra of miRFP718nano (**bottom**). The fluorescence decay was fitted in the whole spectral range with three fluorescence components. The lines in the graph show a spline connection line not supported by further information to guide the eye.

The absorption spectra exhibit the typical maximum at 645 nm for miRFP670nano3 (Figure A3, upper panel) and 690 nm for miRFP718nano (Figure A3, lower panel), without a significant difference between pH 6.0 and 7.4. However, for miRFP670nano3, the band shape of the emission spectrum is rather different from the expectation of a mirrored absorption spectrum (compare Figure A3 top and Figure A4 top). The ground state absorption appears broadened, indicating the existence of two different subpopulations. This agrees with the RR spectroscopic results, including, e.g., two bands at 803 and 816 cm^−1^ in miRFP670nano3, reflecting an equilibrium of two tautomers, one with the proton localized at ring B and the other on ring C. Also, fluorescence spectra and fluorescence decay are practically indistinguishable between pH 6.0 and pH 7.4 for both miRFP670nano3 (Figure A4) and miRFP718nano (Figure A5), with fluorescence maxima at 670 nm for miRFP670nano3 and 718 nm for miRFP718nano. The quantum yields of both proteins are 18.5% and 5.6%, respectively [14]. The fluorescence lifetimes were determined by employing a global fit with three fluorescence components in the whole spectral range between 600 and 800 nm (Appendix B). The resulting decay-associated spectra (DAS) are shown in Figure 4.

The dominating fluorescence emission of mIRFP670nano3 shows a lifetime of 1.73 ns (blue triangles in Figure 4). The emission spectrum (Figure A4, top) has the typical shape of a single chromophore with an emission maximum of 670 nm and a phonon sideband around 700 nm. In the DAS, however, at 700 nm, the peak of a second decay component with a 560 ps lifetime and smaller amplitude is found with a rather broad spectrum between 650 and 750 nm (black squares). The fast 90–100 ps component (red circles) displays a negative amplitude in the whole spectral range.

For miRFP718nano (Figure 4), the dominating fluorescence component seems to be broader than a single Gaussian emitter, without resolved phonon sidebands and with a lifetime of 280 ps and fluorescence maximum at 718 nm (black squares). The long fluorescence component of 1.0–1.1 ns has a very small amplitude only (blue triangles). The red circles show a continuous negative amplitude with 20 ps, which is at the resolution limit. Essentially, the same results were obtained from measurements at pH 6.0 for both variants.

**Figure 5:**
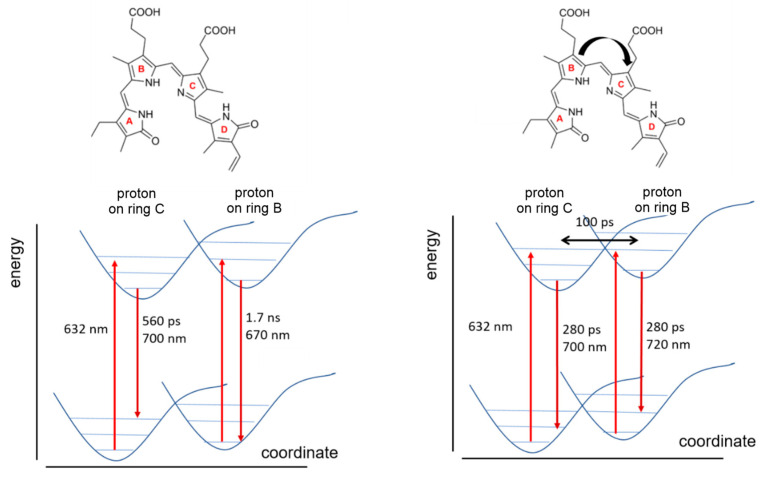
Scheme for intramolecular relaxation after excitation that indicates the dynamics after excitation with 632 nm laser light (red arrow) and two excited states that are populated after vibrational relaxation of the excited state with two different site energies and corresponding rate constants for radiative emission (dark red arrows), nonradiative emission and transition between both states for mIRFP670nano3 (**left**) and miRFP718nano (**right**).

We used the model of a double well potential (Figure 5) to simulate the DAS measured for miRFP670nano3 and mIRFP718nano (Figure 4). The dynamics in the two excited states, as illustrated in Figure 5, are calculated as described in ref. [8] to obtain the observed fluorescence decay components (Equations (A3) and (A4)).

**Figure A6:**
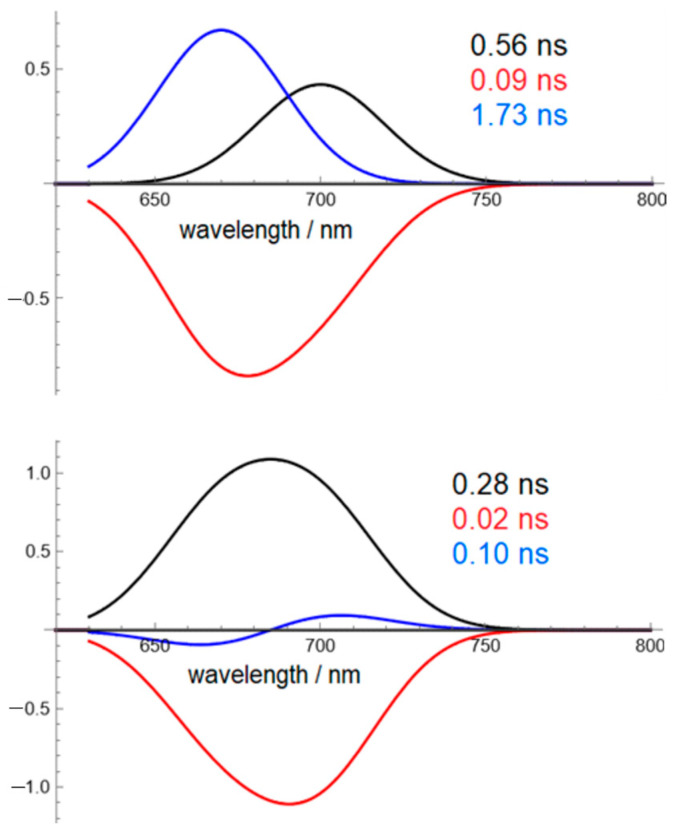
Simulation of the decay-associated spectra (Figure 4) according to the formalism in detail explained in ref. [8] and the excited state model shown in Figure 5.

It is assumed that photon absorption (λ_exc_ = 632 nm) leads to a higher vibronic level in the double well potential of the electronically excited state S_1_, followed by a fast internal conversion to the vibrational ground state of S_1_. This results in a population distribution in two minima of the S_1_ potential curve. As a consequence, there are, in principle, two decays to the corresponding two S_0_ sub-states, each associated with different rate constants for radiative and nonradiative emissions. These decays may be modulated depending on the transition time (*t_ex_*) between the S_1_ minima. For mIRFP718, we postulate an exchange between the S_1_ minima that is fast compared to the excited state lifetime (Figure 5, right), such that different fluorescence decays cannot be resolved in the TRF experiments and only one decay component with 280 ps is obtained. The resolution of the experimental set-up implies *t_ex_* ≤ 100 ps. In contrast, the exchange in the S_1_ of miRFP670nano3 is slow, resulting in two decay routes with distinct relaxation times of 560 ps and 1.7 ns (Figure 5, left side). The DAS simulated on the basis of this model (Figure A6) are in agreement with the measured DAS (Figure 4).

## 3. Discussion

The present results show that the BV chromophores in miRFP670nano3 and miRFP718nano adopt a neutral ZZZssa structure in which one of the inner pyrrole rings B or C is deprotonated (Figure 1). Differences between both miRFPnano variants refer to the C-D methine bridge, as already suggested by the crystal structure [28,29]. This is possibly a consequence of the chromophore deprotonation and reflected by the C-D HOOP modes in the RR spectra, which are different from that of the reference protein iRFP713. Furthermore, the dynamics of the tautomeric equilibrium are quite different in miRFP670nano3 and miRFP718nano. In miRFP670nano3, proton transfer between the nitrogen atoms of rings B and C is relatively slow, presumably due to a high transition barrier resulting from the local electrostatics. In contrast, this barrier is considerably lower in miRFP718nano, leading to a proton transfer between rings B and C with much shorter residence times (in the short ps range), as concluded from the exchange broadening of the RR bands. This fast proton exchange is accompanied by rotational movements of the C-D methine bridge, resulting in higher conformational flexibility of the chromophore, as compared to miRFP670nano3.

In this respect, it is interesting to refer to the CBCR RcaE [21,23]. The deprotonated chromophore in the Pg state gives rise to an absorption spectrum that is similar in shape to that of miRFP670nano3. In both cases, there is no rapid proton exchange between rings B and C, as indicated by the narrow RR bands (Figure 2 and Figure 3, and Ref. [23]). This is in contrast to the absorption and broad RR bands of miRFP718nano that reflect a fast proton exchange.

Most remarkably, the different proton transfer dynamics in the ground states of miRFP670nano3 and miRFP718nano are also mirrored in the excited state, as concluded from the time-resolved fluorescence data and its simulation based on a simple two-state model. Accordingly, in miRFP718nano, the two tautomers are equally populated and equilibrated within the short excited state lifetime of 280 ps, whereas in miRFP670nano3, the two tautomers are separately populated within the fluorescence lifetime of the chromophores and decay with 1.73 ns (ca. 670 nm) and 560 ps (ca. 700 nm) without any appreciable interconversion between the two forms. It is proposed that the B-protonated tautomer is associated with a more rigid structure and corresponds to the 1.73 ns component (ca. 670 nm), while the C-protonated tautomer possibly possesses a more flexible structure corresponding to the 560 ps component (ca. 700 nm). However, regardless of the conformational dynamics of the individual tautomers, the faster tautomeric equilibrium in miRFP718nano causes a distinctly higher overall conformational flexibility of the chromophore compared to miRFP670nano3. This may account for the reduction in the total fluorescence quantum yield from 18.5% in miRFP670nano3 to 5.6% in miRFP718nano [14,28,29]. Determining the individual quantum yields of the two fluorescing components in miRFP670nano3 requires knowledge of their relative populations (Appendix B, Figure A6, Equations (A5)–(A9)).

The most surprising result of the present study, however, is the fact that deprotonated BV chromophores give rise to such high fluorescence yields. Deprotonated tetrapyrroles and specifically BV have been widely studied in a solution [38,39], exhibiting fluorescence quantum yields as low as 0.01% [40,41]. Acidification of the solution causes an increase in the lowest energy absorption at ca. 700 nm and the fluorescence quantum yield to 2%. Protonation of the chromophore is associated with a single-bond isomerization from the helical ZZZsss configuration to an extended ZZZ geometry with at least one methine bridge in the anti-conformation [41]. In proteins, bilins are typically protonated. Only the so-called Meta-Rc intermediates that are transiently formed during the photoconversion of phytochromes or CBCRs and a few green-absorbing parent states, such as in RcaE, harbor a deprotonated chromophore [23,25,42,43]. Notably, in phytochromes, Meta-Rc has been alternatively denoted as I_bleached_ in view of the reduced oscillator strength of the lowest electronic transition [15]. Altogether, these observations led to the general acceptance of a causal chain linking chromophore protonation with a strong electronic transition in the red, as well as high fluorescence yields. The present study contradicts this view since the deprotonated chromophores in miRFP670nano3 and miRFP718nano exhibit fluorescence quantum yields that are comparable to those of fluorescence-optimized iRFPs with protonated chromophores. We can further rule out that there is a protonation–deprotonation equilibrium in miRFP670nano3 and miRFP718nano, corresponding to a fluorescing and non-fluorescing species since there are no differences in the RR, absorption, and fluorescence spectra as well as the fluorescence quantum yields between pH 7.4 and 6.0. Hence, instead of protonation, we conclude that the formation of an extended conformation of the chromophore and its conformational stability is essential for high fluorescence.

**Figure 6:**
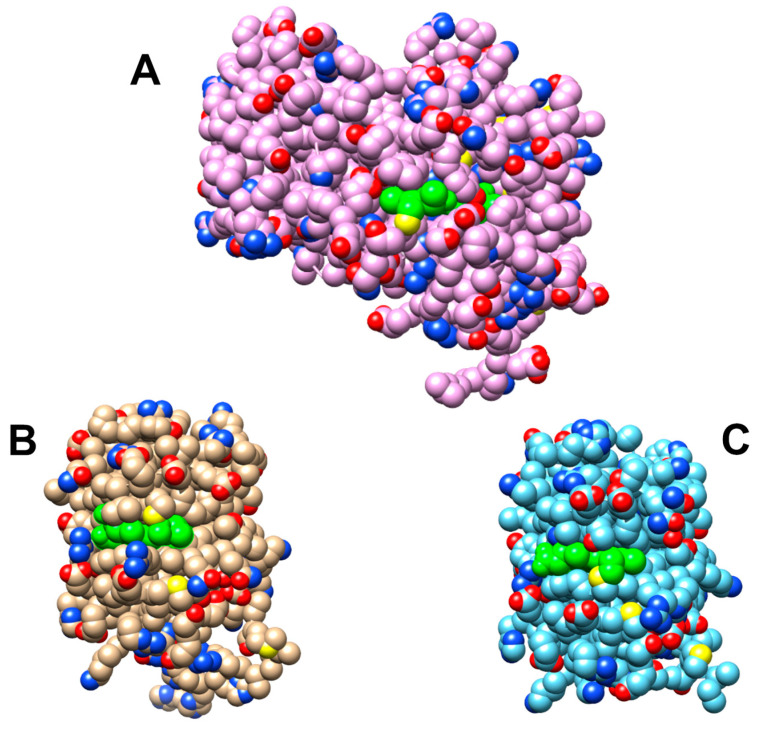
Space filling models of (**A**) bacteriophytochrome P2 from *R. palustris* (PDB entry 4R6L) [33], (**B**) miRFP670nano3 (PDB entry 7LSC) [28], and (**C**) miRFP718nano (PDB entry 7LSD) [29]. The green balls show the chromophore pointing to a higher extent of solvent exposure for miRFP670nano3 and miRFP718nano as compared to RpBphP2 as the wild-type form of iRFP713.

To our knowledge, deprotonated chromophores have not yet been discovered in other iRFPs, raising the question of the origin of this unprecedented observation. The crystallographic structures rule out that—as in the case of the Pg state of RcaE [44,45,46]—a hydrophobic pocket and the lack of an appropriate counter ion stabilize the deprotonated form. Instead, the size reduction of the protein in miRFP670nano3 and miRFP718nano leads a larger portion of the chromophore, specifically of the pyrrole rings B and C, that is exposed to the solution phase to a distinctly larger extent than “classical” iRFPs (Figure 6). Hence, the pK_a_ for the protonation of ring B or C approaches that of BV in solution, which is estimated to be below 2.0 [47].

## 4. Materials and Methods

### 4.1. Protein Expression and Purification

To produce IR fluorescent proteins in *E. coli*, a biliverdin fluorophore is required. Thus, the cDNA coding for human heme oxygenase (accession number P09601 in https://www.uniprot.org/uniprotkb/P09601/entry (accessed on 26 July 2022)), which converts heme into biliverdin, was synthesized (Azenta GmbH, Leipzig, Germany) and first cloned into the expression vector pETDuet™-1 (Merck Millipore GmbH, Darmstadt, Germany) using *Nco*I and *Pac*I restriction sites, resulting in the pETduet1-hHO-1 vector. The cDNA coding for miRFP670nano3 [13,28] and miRFP718nano [14,29] were synthesized (Azenta GmbH, Germany) and subsequently inserted into the pETduet1-hHO-1 vector via *Nco*I and *Not*I restriction sites, resulting in the recombinant vectors for pETDuet-1-miRFP670nano3-hHO-1 and pETDuet-1-miRFP718-hHO-1. Recombinant miRFP670nano3 and miRFP718nano were synthesized as fusion proteins containing two C-terminal tags. The His-tag (6xHis) was used for protein purification by immobilized metal ion chromatography (IMAC) and the c-Myc-tag for Western blot analysis, respectively. The expression of hHO-1 and fluorescent proteins was controlled by the T7 expression system in *E. coli* BL21 (DE3), which was the expression host. The authenticity of cloned sequences in recombinant vectors was confirmed by Sanger sequencing.

Constructed expression vectors (pETDuet-1-mRFP670nano3-hHO-1 and pETDuet-1-mRFP718-hHO-1) were introduced into the expression chemo competent *E. coli* BL21 strain (DE3) by heat shock. Bacteria were spread on Luria–Bertani (LB) agar plates containing carbenicillin (100 µg/mL). After 20 h of incubation at 37 °C, single colonies were picked and streaked into fresh LB plates supplemented with carbenicillin and incubated at 37 °C. Cells from streaked plates were cultured in 800 mL of carbenicillin containing LB and were harvested by a Beckman Coulter Avanti JXN-26 centrifuge with a JA-10 rotor at 11,330× *g* and 4 °C and stored at −80 °C (Beckman Coulter, Brea, CA, USA).

One gram of *E. coli* cell pellets was suspended in 5 mL of a binding buffer (50 mM NaH_2_PO_4_, 300 mM NaCl, pH 8.0). The cell suspension was homogenized by the one-shot cell disrupter at 20 kPsi pressure (Constant systems, Daventry, UK). The resulting cell lysate was centrifuged at 50,000× *g* for 30 min at 4 °C using a JS24-30 rotor and Aventi JXN-26 centrifuge to remove the cell debris (Beckman Coulter, Brea, CA, USA). The clarified cell extract was collected and filtered through a 0.22 µm filter membrane. The filtered extract containing His-tagged fluorescent proteins was loaded into a HisTrap-HP-1ml (Cytiva, Uppsala, Sweden) attached to an ÄKTA prime plus chromatography system. The column was then washed with a washing buffer (50 mM NaH_2_PO_4_, 300 mM NaCl, 35 mM imidazole, and pH 8.0). Finally, the bound fluorescent proteins were eluted using an elution buffer (50 mM NaH_2_PO_4_, 300 mM NaCl, 125 mM imidazole, and pH 8.0). The eluted protein solution was dialyzed against PBS at 4 °C for 24 h using SERVAPOR 6 dialysis tubing, MWCO 6000-8000 RC, and a diameter of 16 mm (SERVA Electrophoresis GmbH, Heidelberg, Germany), and then concentrated using a Pierce^TM^ Protein Concentrator PES 10K MWCO (Thermo Scientific, Dreieich, Germany). Purified recombinant proteins were analyzed by SDS-PAGE and stained with Coomassie blue and zinc staining.

Proteins were diluted by using the SDS-PAGE sample buffer four times and incubated for 5 min at 95 °C. The resulting solutions were separated by reducing SDS-PAGE (15% polyacrylamide). To image zinc-induced fluorescence [48], SDS-PAGE gels were stained with 100 mM ZnSO_4_ solution for 15 min at room temperature. Gels were visualized using the ChemiDoc MP Imaging System (Bio-rad Laboratories, Feldkirchen, Germany). ZnSO_4_-treated gel was then stained with a Coomassie stain solution.

### 4.2. Spectroscopy

Protein suspensions were concentrated from buffered solutions in H_2_O (pH 7.4) or D_2_O (pD 7.4) to ca. 10 mM for RR experiments, whereas for absorption and fluorescence spectroscopy, concentrations of 10 µM or less were used. RR measurements were performed using a Bruker Fourier-transform MultiRAM spectrometer equipped with a Ramanscope III and an Nd-YAG cw laser for 1064 nm excitation (line width 1 cm^−1^) (Bruker, Karlsruhe, Germany). The spectrometer was coupled to a nitrogen-cooled cryostat from Resultec (Linkam, Salfords, UK). All spectra of the samples were recorded in frozen solution at ca. 90 K with a laser power at the sample of 690 mW and a typical accumulation time of one hour. Potential laser-induced damage of the proteins could be ruled out since a comparison of RR spectra before and after a series of measurements did not reveal any changes.

Absorption spectra were recorded with a USB-650-UV-VIS absorption spectrometer and OceanView (Ocean Optics, Ostfildern, Germany). Fluorescence spectra were conducted using a 1 cm quartz silica cuvettes (Thorlabs Inc., Bergkirchen, Germany), employing a commercial USB-connected fluorometer system with a CCD array (black comet, CXR-SR UV/VIS/NIR, Scientific Instruments, Gilching, Germany) delivering a spectral resolution of 2 nm. A 632 nm pulsed-laser diode (PDL-600, Becker & Hickl, Berlin, Germany) delivering 80 ps FWHM pulses, driven at a repetition rate of 20 MHz, was used for excitation. 

Spectrally resolved fluorescence decay curves were recorded from a 10 µM protein solution in a C3 buffer at room temperature. Measurements were conducted in 300 µL quartz silica cuvettes (Thorlabs Inc., Bergkirchen, Germany) employing a Hamamatsu R5900 16-channel multi-anode photomultiplier tube (PMT) with 16 separate output elements and a common cathode and dynode system (PML-16C, Becker & Hickl, Berlin, Germany). The polychromator was equipped with a 300 lines/mm grating, resulting in a spectral bandwidth of the PML-16C of 12.5 nm/channel. A 632 nm pulsed laser diode (PDL-600, Becker&Hickl, Berlin, Germany), delivering 80 ps FWHM pulses at a repetition rate of 20 MHz, was used for excitation. The fluorescence was observed via a 632 nm long pass filter (F76-631, AHF Analysentechnik, Tübingen, Germany). Fluorescence data were analyzed and the DAS were calculated and simulated, as described in detail previously [8].

## 5. Conclusions

The two minimal iRFPs, miRFP670nano3 and miRFP718nano, studied in this work share an unprecedented property of its chromophore; that is, the deprotonation of one of the inner pyrrole rings B or C. This seems to be the consequence of the partial solvent exposure of covalently bound BV. Nevertheless, the fluorescence quantum yields are high (with 18.5% in miRFP670nano3 and 5.6% in miRFP718nano), implying that the protonation of tetrapyrroles (cationic form) is not a prerequisite for a high fluorescence yield. Instead, an extended geometry (e.g., ZZZssa) as the preferred tetrapyrrole structure in proteins but thermodynamically less stable in a solution may be a crucial parameter that favors fluorescence. The main difference between both proteins is the proton dynamics. In miRFP670nano3, the transition between the B-protonated and C-protonated tautomer is slow compared to the excited state lifetime, whereas in miRFP718nano, proton exchange is very fast in both the ground and excited state, presumably due to a higher flexibility of the tetrapyrrole geometry. It is, therefore, tempting to ascribe the distinctly higher fluorescence quantum yield in miRFP670nano3 to this structural difference.

## Data Availability

The original contributions presented in the study are included in the article, further inquiries can be directed to the corresponding authors.

## Appendix A

Appendix A includes additional RR and fluorescence data. RR spectra show the effect of H/D exchange in a wide spectral range (Figure A1), complementary to Figure 2. Figure A2 demonstrates that RR spectra do not change between pH 7.4 and 6.0. This is also true for the absorption and fluorescence spectra as well as the fluorescence decay curves in Figure A3, Figure A4 and Figure A5.

## Appendix B

Based on the model and the simulated DAS (Figure A6), we may rationalize the experimental fluorescence quantum yields.

In miRFP718nano, the two tautomers are equilibrated within the short-excited state lifetime. Thus, there is essentially only one fluorescence component resolved in the DAS (Figure 4) with a quantum yield of 5.6%, according to

(A1) $$\Phi =\frac{k_{F}}{k_{F}+k_{nr}}=5.6\%$$

where *k_F_* and *k_nr_* are the rate constants for the radiative and nonradiative decay, respectively. With the observed fluorescence decay time

τ = 1/*k* = 1/(*k_F_* + *k_nr_*)
(A2)

of 280 ps one then obtains $k_{nr}=3.4\cdot 10^{9}\;s^{-1}$ and $k_{F}=2\cdot 10^{8}\;s^{-1}$. The time-dependent fluorescence is then described by

(A3) $$F\left(t\right)=F_{0}\exp\left(-kt\right)=F_{0}\exp\left(-\frac{t}{280\;ps}\right)$$

The fluorescence of miRFP670nano3 involves two components “1” and “2”

(A4) $$F\left(t\right)=F_{01}\exp\left(-k_{1}t\right){+F}_{02}\exp\left(-k_{2}t\right)=F_{01}\exp\left(-\frac{t}{560\;ps}\right)+F_{02}\exp\left(-\frac{t}{1.7\;ns}\right)$$

Equations (A1) and (A2) now hold separately for each of these components:

(A5) $$\Phi_{1,2}=\frac{k_{F1,2}}{k_{F1,2}+k_{nr1,2}}$$

(A6) $$\tau_{1,2}=\frac{1}{k_{1,2}}=\frac{1}{k_{F1,2}+k_{nr1,2}}$$

Therefore, from the measured fluorescence rate constants *k*_1,2_ and the corresponding amplitudes *F*_01_ and *F*_02_, there is a unique solution for all individual radiative and nonradiative decay rates of both configurations of miRFP670nano3.

The ratio of the different populations of the individual components *n*_1_/*n*_2_ is calculated from the ratios of the initial amplitudes of the DAS components in Figure 4

(A7) $$\frac{n_{1}}{n_{2}}=\frac{F_{01}}{F_{02}}\approx \frac{5}{7}$$

while the quantum yield is the product of time constants and amplitudes and, therefore, the ratio of the quantum yields of both configurations can be derived from the TRF measurements according to

(A8) $$\frac{\Phi_{1}}{\Phi_{2}}=\frac{F_{01}\tau_{1}}{F_{02}\tau_{2}}=0.23$$

The known total fluorescence quantum yield Φ*_tot_* of 18.5% [11] is related to the individual quantum yields according to the population-weighted contribution of the quantum yields of both configurations:

(A9) $$\Phi_{tot}=\frac{n_{1}\Phi_{1}+n_{2}\Phi_{2}}{n_{1}+n_{2}}=18.5\%$$

Thus, Equations (A5)–(A7) yield

$$\Phi_{1}=6.3\%\;\Phi_{2}=27.2\%$$

$$k_{F1}=1.13\cdot 10^{8}\;s^{-1}\;k_{nr1}=1.67\cdot 10^{9}\;s^{-1}$$

$$k_{F2}=1.57\cdot 10^{8}\;s^{-1}\;k_{nr2}=4.2\cdot 10^{8}\;s^{-1}$$

At present, we are not able to assign the individual components to the B- and C-protonated tautomers. In this context, future calculations of the excited state properties may provide additional information and thus may guide the stabilization of the tautomer with the highest fluorescence yield.

## References

[1] Daetwyler S., Fiolka R.P. (2023). Light-Sheets and Smart Microscopy, an Exciting Future Is Dawning. Commun. Biol..

[2] Miller D.R., Jarrett J.W., Hassan A.M., Dunn A.K. (2017). Deep Tissue Imaging with Multiphoton Fluorescence Microscopy. Curr. Opin. Biomed. Eng..

[3] Karasev M.M., Stepanenko O.V., Rumyantsev K.A., Turoverov K.K., Verkhusha V.V. (2019). Near-Infrared Fluorescent Proteins and Their Applications. Biochemistry.

[4] Shcherbakova D.M. (2021). Near-Infrared and Far-Red Genetically Encoded Indicators of Neuronal Activity. J. Neurosci. Methods.

[5] Li J., Dong Y., Wei R., Jiang G., Yao C., Lv M., Wu Y., Gardner S.H., Zhang F., Lucero M.Y. (2022). Stable, Bright, and Long-Fluorescence-Lifetime Dyes for Deep-Near-Infrared Bioimaging. J. Am. Chem. Soc..

[6] Oliinyk O.S., Chernov K.G., Verkhusha V.V. (2017). Bacterial Phytochromes, Cyanobacteriochromes and Allophycocyanins as a Source of near-Infrared Fluorescent Probes. Int. J. Mol. Sci..

[7] Shen Y., Lai T., Campbell R.E. (2015). Red Fluorescent Proteins (RFPs) and RFP-Based Biosensors for Neuronal Imaging Applications. Neurophotonics.

[8] Schmitt F.J., Mehmood A.S., Tüting C., Phan H.T., Reisdorf J., Rieder F., Ghane Golmohamadi F., Verma R., Kastritis P.L., Laufer J. (2024). Effect of Molecular Dynamics and Internal Water Contact on the Photophysical Properties of Red PH-Sensitive Proteins. Biochemistry.

[9] Fischer A.J., Lagarias J.C. (2004). Harnessing Phytochrome’s Glowing Potential. Proc. Natl. Acad. Sci. USA.

[10] Wagner J.R., Zhang J., von Stetten D., Günther M., Murgida D.H., Mroginski M.A., Walker J.M., Forest K.T., Hildebrandt P., Vierstra R.D. (2008). Mutational Analysis of Deinococcus Radiodurans Bacteriophytochrome Reveals Key Amino Acids Necessary for the Photochromicity and Proton Exchange Cycle of Phytochromes. J. Biol. Chem..

[11] Toh K.C., Stojković E.A., Van Stokkum I.H.M., Moffat K., Kennis J.T.M. (2010). Proton-Transfer and Hydrogen-Bond Interactions Determine Fluorescence Quantum Yield and Photochemical Efficiency of Bacteriophytochrome. Proc. Natl. Acad. Sci. USA.

[12] Matlashov M.E., Shcherbakova D.M., Alvelid J., Baloban M., Pennacchietti F., Shemetov A.A., Testa I., Verkhusha V.V. (2020). A Set of Monomeric Near-Infrared Fluorescent Proteins for Multicolor Imaging across Scales. Nat. Commun..

[13] Oliinyk O.S., Shemetov A.A., Pletnev S., Shcherbakova D.M., Verkhusha V.V. (2019). Smallest Near-Infrared Fluorescent Protein Evolved from Cyanobacteriochrome as Versatile Tag for Spectral Multiplexing. Nat. Commun..

[14] Oliinyk O.S., Pletnev S., Baloban M., Verkhusha V.V. (2023). Development of Bright Red-Shifted MiRFP704nano Using Structural Analysis of MiRFPnano Proteins. Protein Sci..

[15] Sineshchekov V.A. (1995). Photobiophysics and Photobiochemistry of the Heterogeneous Phytochrome System. Biochim. Biophys. Acta.

[16] Liu F., Hu H., Deng M., Xiang Z., Guo Y., Guan X., Li D., Hu Q., Lei W., Peng H. (2022). A Bright Monomeric Near-Infrared Fluorescent Protein with an Excitation Peak at 633 Nm for Labeling Cellular Protein and Reporting Protein-Protein Interaction. ACS Sens..

[17] Hall C., von Grabowiecki Y., Pearce S.P., Dive C., Bagley S., Muller P.A.J. (2021). IRFP (near-Infrared Fluorescent Protein) Imaging of Subcutaneous and Deep Tissue Tumours in Mice Highlights Differences between Imaging Platforms. Cancer Cell Int..

[18] Yoshihara S., Ikeuchi M. (2004). Phototactic Motility in the Unicellular Cyanobacterium *Synechocystis* sp. PCC 6803. Photochem. Photobiol. Sci..

[19] Escherichia P., Yoshihara S., Shimada T., Matsuoka D., Zikihara K., Kohchi T. (2006). Reconstitution of Blue—Green Reversible Photoconversion of a Cyanobacterial. Biochemistry.

[20] Rockwell N.C., Martin S.S., Lagarias J.C. (2012). Red/Green Cyanobacteriochromes: Sensors of Color and Power. Biochemistry.

[21] Hirose Y., Rockwell N.C., Nishiyama K., Narikawa R., Ukaji Y., Inomata K., Lagarias J.C., Ikeuchi M. (2013). Green/Red Cyanobacteriochromes Regulate Complementary Chromatic Acclimation via a Protochromic Photocycle. Proc. Natl. Acad. Sci. USA.

[22] Sato T., Kikukawa T., Miyoshi R., Kajimoto K., Yonekawa C., Fujisawa T., Unno M., Eki T., Hirose Y. (2019). Protochromic Absorption Changes in the Two-Cysteine Photocycle of a Blue/Orange Cyanobacteriochrome. J. Biol. Chem..

[23] Osoegawa S., Miyoshi R., Watanabe K., Hirose Y., Fujisawa T., Ikeuchi M., Unno M. (2019). Identification of the Deprotonated Pyrrole Nitrogen of the Bilin-Based Photoreceptor by Raman Spectroscopy with an Advanced Computational Analysis. J. Phys. Chem. B.

[24] Ishizuka T., Narikawa R., Kohchi T., Katayama M., Ikeuchi M. (2007). Cyanobacteriochrome TePixJ of Thermosynechococcus Elongatus Harbors Phycoviolobilin as a Chromophore. Plant Cell Physiol..

[25] Velazquez Escobar F., Utesch T., Narikawa R., Ikeuchi M., Mroginski M.A., Gärtner W., Hildebrandt P. (2013). Photoconversion Mechanism of the Second GAF Domain of Cyanobacteriochrome AnPixJ and the Cofactor Structure of Its Green-Absorbing State. Biochemistry.

[26] Hirose Y., Shimada T., Narikawa R., Katayama M., Ikeuchi M. (2008). Cyanobacteriochrome CcaS Is the Green Light Receptor That Induces the Expression of Phycobilisome Linker Protein. Proc. Natl. Acad. Sci. USA.

[27] Fushimi K., Miyazaki T., Kuwasaki Y., Nakajima T., Yamamoto T., Suzuki K., Ueda Y., Miyake K., Takeda Y., Choi J.H. (2019). Rational Conversion of Chromophore Selectivity of Cyanobacteriochromes to Accept Mammalian Intrinsic Biliverdin. Proc. Natl. Acad. Sci. USA.

[28] Oliinyk O.S., Baloban M., Clark C.L., Carey E., Pletnev S., Nimmerjahn A., Verkhusha V.V. (2022). Single-Domain near-Infrared Protein Provides a Scaffold for Antigen-Dependent Fluorescent Nanobodies. Nat. Methods.

[29] Oliinyk O.S., Ma C., Pletnev S., Baloban M., Taboada C., Sheng H., Yao J., Verkhusha V.V. (2023). Deep-Tissue SWIR Imaging Using Rationally Designed Small Red-Shifted near-Infrared Fluorescent Protein. Nat. Methods.

[30] Filonov G.S., Piatkevich K.D., Ting L.M., Zhang J., Kim K., Verkhusha V.V. (2011). Bright and Stable Near-Infrared Fluorescent Protein for in Vivo Imaging. Nat. Biotechnol..

[31] Hildebrandt P. (2023). Vibrational Spectroscopy of Phytochromes. Biomolecules.

[32] Velazquez Escobar F., Hildebrandt T., Utesch T., Schmitt F.J., Seuffert I., Michael N., Schulz C., Mroginski M.A., Friedrich T., Hildebrandt P. (2013). Structural Parameters Controlling the Fluorescence Properties of Phytochromes. Biochemistry.

[33] Yang X., Stojković E.A., Ozarowski W.B., Kuk J., Davydova E., Moffat K. (2015). Light Signaling Mechanism of Two Tandem Bacteriophytochromes. Structure.

[34] Kneip C., Hildebrandt P., Schlamann W., Braslavsky S.E., Mark F., Schaffner K. (1999). Protonation State and Structural Changes of the Tetrapyrrole Chromophore during the Pr → Pfr Phototransformation of Phytochrome: A Resonance Raman Spectroscopic Study. Biochemistry.

[35] López M.F., Dahl M., Escobar F.V., Bonomi H.R., Kraskov A., Michael N., Mroginski M.A., Scheerer P., Hildebrandt P. (2022). Photoinduced Reaction Mechanisms in Prototypical and Bathy Phytochromes. Phys. Chem. Chem. Phys..

[36] Salewski J., Escobar F.V., Kaminski S., Von Stetten D., Keidel A., Rippers Y., Michael N., Scheerer P., Piwowarski P., Bartl F. (2013). Structure of the Biliverdin Cofactor in the Pfr State of Bathy and Prototypical Phytochromes. J. Biol. Chem..

[37] Strehlow H., Wagner I., Hildebrandt P. (1983). Chemical Exchange and Raman Line Broadening. the Rate of Protolysis of Nitric Acid. Berichte Der Bunsenges./Phys. Chem. Chem. Phys..

[38] Taniguchi M., Lindsey J.S. (2018). Database of Absorption and Fluorescence Spectra of >300 Common Compounds for Use in PhotochemCAD. Photochem. Photobiol..

[39] Taniguchi M., Lindsey J.S. (2023). Absorption and Fluorescence Spectra of Open-Chain Tetrapyrrole Pigments–Bilirubins, Biliverdins, Phycobilins, and Synthetic Analogues. J. Photochem. Photobiol. C Photochem. Rev..

[40] Braslavsky S.E., Holzwarth A.R., Lehner H., Schaffner K. (1978). The Fluorescence of Biliverdin Dimethyl Ester. Helv. Chim. Acta.

[41] Braslavsky S.E., Holzwarth A.R., Schaffner K. (1983). Solution Conformations, Photophysics, and Photochemistry of Bile Pigments; Bilirubin and Biliverdin, Dimethyl Esters and Related Linear Tetrapyrroles. Angew. Chemie Int. Ed. Engl..

[42] Mizutani Y., Kitagawa T., Tokutomi S., Aoyagi K., Horitsu K. (1991). Resonance Raman Study on Intact Pea Phytochrome and Its Model Compounds: Evidence for Proton Migration during the Phototransformation. Biochemistry.

[43] Borucki B., von Stetten D., Seibeck S., Lamparter T., Michael N., Mroginski M.A., Otto H., Murgida D.H., Heyn M.P., Hildebrandt P. (2005). Light-Induced Proton Release of Phytochrome Is Coupled to the Transient Deprotonation of the Tetrapyrrole Chromophore. J. Biol. Chem..

[44] Margulies L., Toporowicz M. (1984). Resonance Raman Study of Model Compounds of the Phytochrome Chromophore. 2. Biliverdin Dimethyl Ester. J. Am. Chem. Soc..

[45] Jiang H.J., Underwood T.C., Bell J.G., Ranjan S., Sasselov D., Whitesides G.M. (2017). Mimicking Lighting-Induced Electrochemistry on the Early Earth. Proc. Natl. Acad. Sci. USA.

[46] Nagae T., Unno M., Koizumi T., Miyanoiri Y., Fujisawa T., Masui K., Kamo T., Wada K., Eki T., Ito Y. (2021). Structural Basis of the Protochromic Green/Red Photocycle of the Chromatic Acclimation Sensor RcaE. Proc. Natl. Acad. Sci. USA.

[47] Nagae T., Fujita Y., Tsuchida T., Kamo T., Seto R., Hamada M., Aoyama H., Sato-Tomita A., Fujisawa T., Eki T. (2024). Green/Red Light-Sensing Mechanism in the Chromatic Acclimation Photosensor. Sci. Adv..

[48] Raps S. (1990). Differentiation between Phycobiliprotein and Colorless Linker Polypeptides by Fluorescence in the Presence of ZnSO_4_. Plant Physiol..

